# New Appliances and Things Medical

**Published:** 1898-09-10

**Authors:** 


### NEW APPLIANCES AND THINGS MEDICAL.

[We shall be glad to receive, at our Office 28 & 29, Southampton Street, Strand, London, W.C., from the manufacturers, specimens of all new preparations and appliances which may be brought out from time to time.]

### SCOTCH WHISKY.

(DANIEL CRAWFORD AND SON, 81, QUEEN STREET, GLASGOW.)

We have received a sample of this very fine old Scotch whisky. As the result of our examination, we regard it as a very finely matured spirit, of pleasant flavour and first-rate quality. As a whisky for invalids and for general consumption we recommend it highly to the [illegible]ce of our readers.

